# Infrared thermographic imaging as a promising on-farm method to estimate parasite load in dairy goats

**DOI:** 10.1016/j.vas.2025.100506

**Published:** 2025-09-12

**Authors:** M. Bernau, T. Schilling, H. Eßlinger, L.E. Hoelzle, S.A. Goth

**Affiliations:** a Hochschule für Wirtschaft und Umwelt Nürtingen-Geislingen, Fakultät Agrarwirtschaft, Neckarsteige 6-10, 72622 Nürtingen, Germany; b Universität Hohenheim, Institut für Nutztierwissenschaften, Fachgebiet Infektions- und Umwelthygiene bei Nutztieren, Garbenstr. 30, 70599 Stuttgart, Germany

**Keywords:** Thermographic imaging, Dairy goats, Faecal egg count, Health indicators, Parasite load

## Abstract

Faecal egg count (FEC) evaluation is the method of choice to detect parasite load in individuals, but it cannot be used directly on farm. Clinical parameters like body condition scores (BCS), body weight (BW) and milk yield (MY) have been described in this context, but all fail due to a low correlation with FEC. The present study evaluated the utility of clinical parameters, linear body measurements, and infrared thermographic imaging (IRT) in eight dairy goat herds in Germany as potential methods for estimating FEC categories. A total of 893 dairy goats comprising German Fawn Goats (GFG) and German White Goats (GWG) were examined up to six times during three consecutive lactation periods. FEC was determined using individual faecal samples, analysed via the McMaster method. Based on eggs per gram of faeces (EPG), mean FEC across all examinations was categorized into three groups: low (Category 1: ≤500 EPG), medium (Category 2: >500 to ≤1500 EPG), and high (Category 3: >1500 EPG). Statistical analyses were conducted using SAS Studio to identify factors influencing FEC. Significant differences were observed between the two breeds.

In GFG, animals in Category 3 exhibited significantly lower BCS, BW, chest circumference, and MY compared to those in Category 1. IRT measurements also showed significant differences across FEC categories, with higher IRT values observed in goats with higher FEC. These findings suggest that IRT has potential as a non-invasive, on-farm method for assessing parasitic load. However, further studies are necessary to validate its practical application as a direct health monitoring method.

## Highlights

• Study represents estimation of parasite load using clinical parameters, linear body measurements and infrared thermographic (IRT) measurements.
• A total of 893 dairy goats (German Fawn goat and German White goat) were examined up to six times during three consecutive lactation periods.
• Significant differences between breeds exist.
• Significant effects were evaluated using IRT measurements compared to the FEC categories, with higher IRT´s in higher FEC category.

## 1 Introduction

Parasite load is an creasing concern in small ruminant health, as it compromises animal welfare, reduces productivity, and increases management costs, thereby diminishing economic returns (Charlier et al., 2020; Mehmood et al., 2017). Alarming levels of anthelminthic resistances (Kaplan & Vidyashankar, 2012; Sander et al., 2024; Voigt et al., 2023) lead to the need of targeted selected treatment and with this the identification of infected animals is the major goal for treatment purposes.

The McMaster method remains the most widely used standard quantitative technique for evaluating faecal egg count (FEC) to identify parasitic infections (Gordon & Whitlock, 1939; Whitlock, 1948). This method is based on the examination of a defined amount of either individual or pooled faecal samples.

But, to detect the infected animal on farm, clinical signs are of major interest. Some are already described in the literature like the bottle jaw, low body condition scores, faecal soiling (Bath & van Wyk, 2009), body weight changes (Amarante et al., 2004; Van Wyk et al., 2006) or a decrease in milk yield (Hoste et al., 2002). For haematophagous parasites the FAMACHA© scoring is recommended (Bath & van Wyk, 2009; Şahin et al., 2021; Vatta et al., 2001). But, as shown in sheep, clinical signs correlate low or not to FEC, which does not result in the identification of animals having a high parasite load based on clinical signs solely (Sajovitz et al., 2024).

Therefore, there is a need of a practical method, which can be used on farm to identify animals with high parasite load. Maybe other parameters can be linked to parasite load, too? In breeding purposes, linear measurements are widely used to describe the animal in more detail and with higher accuracy (Herold et al., 2018; Lange et al., 2018). Some of these measurements can be used on farm as well, as the study of Silva et al. (2024) demonstrated by estimating calves weight using linear measurements. But whether linear measurements can be used to estimate health parameters in terms of parasitic load is still unclear.

Additionally, newly used imaging methods like infrared thermographic imaging (IRT) can be used to detect elevated or lowered temperatures which might be used as key indicators for infectious diseases (Mota-Rojas et al., 2022), like shown in dairy cows for subclinical mastitis (Oliveira et al., 2022) or in terms of ectoparasite detection (Cortivo et al., 2016). Changes in thermal windows, detectable via IRT (Mota-Rojas et al., 2021), might be a possibility to gain information about parasite load in animals, as parasitic load results in a wide range of effects (Hawkins, 1993).

Therefore, the aim of this study was to estimate parasite load (FEC) in dairy goats by integrating established clinical indicators with novel methods, with the goal of developing a practical approach for assessing individual FEC directly on-farm.

## 2 Material & methods

### 2.1 Study design

Between spring 2022 to spring 2024, a total of 893 dairy goats from eight German farms were examined one to six times over three years – during the beginning, the middle and the end of the lactation period (spring: March to beginning of June; summer: end of June to August; autumn/winter: October to November).

The study included two breeds: German Fawn goats (GFG; *n* = 558) and German White goats (GWG; *n* = 335). Goats were selected based on performance, with four animals of each yield group (high, middle, and low; in each case in relation to the herd performance), along with one lactating daughter each. This was done to reflect the overall herd distribution in terms of yield groups for each farm.

### 2.2 Methods

At examination day, a number of maximum 24 goats were selected from each herd (still having visual contact to the herd) by their owner. All examinations were conducted by a single trained investigator to ensure consistency. At examination day, all goats were kept indoor. This ensures that differences in thermography imaging were not caused by solar radiation, wind or weather conditions. Ambient temperatures and humidity in the stable were collected in the beginning and the end of each examination day, using a testo 174 H data logger (Testo SE & Co. KG, Germany). Temperatures and humidity’s differed only slightly during examinations within a season (mean values: spring (12.3 ± 4.0 °C; 62.0 ± 7.5 % humidity), summer (22.0 ± 1.6 °C; 52.9 ± 8.4 % humidity), autumn/winter (15.9 ± 3.9 °C; 70.8 ± 6.6 % humidity)).

**Fig. 1:**
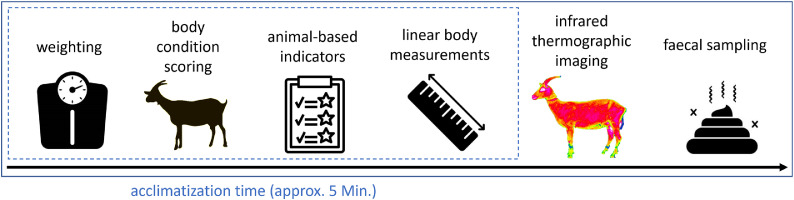
Examination schedule. Dotted area represents the acclimatization time prior infrared thermographic imaging⁎.

The following data was assessed for each goat (Fig. 1 demonstrates the examination schedule):

1. **Weight, Body Condition Score (BCS) & animal-based indicators:** The goat was weighted on a platform-scale (myWeight, VHD-3). After weighting, the body condition score (BCS) was examined, using the described lumbal and sternal score from 1 to 5 according to Leeb et al. (2007). Additionally, four animal-based indicators (nasal discharge, eye discharge, faecal soiling, and coat condition) were scored from 0 (absence; without alterations) to 1 (presence; with alterations), according to AWIN (2015) and Sporkmann and Georg (2018) (see Table 1).

**Table 1:** Parameter description for the used animal-based indicators.

Parameter	Description
**Nasal discharge**	Scoring according to AWIN (2015) Score 0= absence (not one-sided either) Score 1= presence, as mucous or purulent discharge
**Eye discharge**	Scoring according to AWIN (2015) Score 0= absence (not one-sided either) Score 1= presence
**Faecal soiling**	Scoring according to Sporkmann and Georg (2018) Score 0= tail is free from faeces Score 1= faecal remains are found on the tail
**Coat condition**	Scoring according to Sporkmann and Georg (2018) Score 0= healthy coat, which is smooth and shiny on the body Score 1= poor looking coat, dull, lustreless and brittle
2. **Linear measurements:** Two linear measurements were taken directly on the animal using a scale: chest circumference (circumference behind the elbow) and belly circumference (widest circumference at the last rip).
3. **Infrared thermographic imaging:**
  **Image acquisition:**
  Infrared thermographic images were taken with a handheld VarioCAM^(R)^ HDx 645 camera (Infratec, Dresden, Germany; 640 × 480 IR-pixel, automatic calibration, 0.02 K thermal sensitivity, -40 °C to 2000 °C temperature range, ±1 % within the ambient temperature range) and an adjusted emissivity of 0.98. Images were taken from the left and right body side of the goat, from the front view and from the hind view (see Fig. 2). During imaging, all goats were positioned in front of a green screen in order to avoid affection from the background, as recommended by Velasco-Bolanos et al. (2021). The camera was positioned with a distance to the goat from approximately 4.5 m, aligned at the animal´s height.

**Fig. 2:**
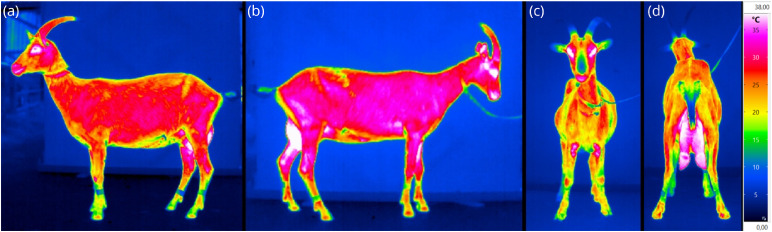
Infrared thermographic images of horned goats: one goat displayed in left side view (a), one goat displayed in right side view (b), another goat displayed from front view (c) and from hind view perspective (d).
  **Image analyses:**
  Images were analyzed with a fixed temperature scale (from 0 to 38 °C). Two kinds of image analyses were performed:
  1) by measuring infrared temperatures using the IRBIS®3pro software (InfraTec GmbH, Dresden, Germany) for eye (IRT_eye_temp) and udder (IRT_udder_temp) (see Fig. 3*a* + *b*): To assess these temperatures, images were analysed as follows:
    (a) for IRT_eye_temp the temperature point at the highest temperature in the inner ocular corner was analysed (side view, left and right side; see Fig. 3a).
    (b) for IRT_udder_temp a temperature line was used, measuring from vulva to beginning of the teat of the left and right udder side, given as mean temperature for the line, separated for each side and pooled (hind view; see Fig. 3b).

**Fig. 3:**
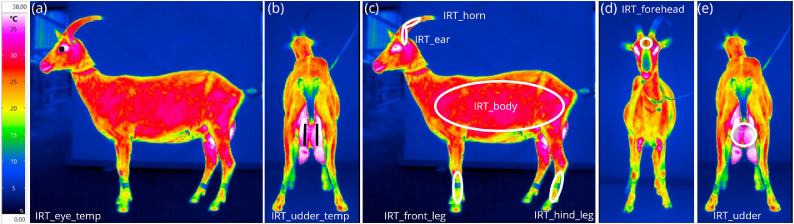
Presentation of infrared thermographic image analyses. (a) measuring eye infrared temperature using the IRBIS®3pro software; (b) measuring udder infrared temperature using the IRBIS®3pro software; (c) areas of interest for the color coded image analysis on the side view image (ear, horn, body, front leg, hind leg); (d) area of interest for the color coded image analysis on the front view image (forehead); (e) area of interest for the color coded image analysis on the hind view image (udder).

and

  2) by using a color-coded scoring on a scale from 1 to 11 (see Fig. 4) for 12 body regions: Color coding was used for the chosen body regions, scoring the main color of the chosen area. Each color results in a number and this could be converted into infrared thermographic temperatures (see Fig. 4). The following regions were scored:
    ■ scores (*n* = 5 per side) taken from the left and the right body side (Fig. 3c): ear (IRT_ear), horn (IRT_horn), body (IRT_body), lower front (IRT_front leg) and lower hind leg (IRT_hind leg).
    ■ score taken from the front view perspective (Fig. 3d): forehead (IRT_forehead).
    ■ score taken from the hind view perspective (Fig. 3e): udder (IRT_udder).

**Fig. 4:**
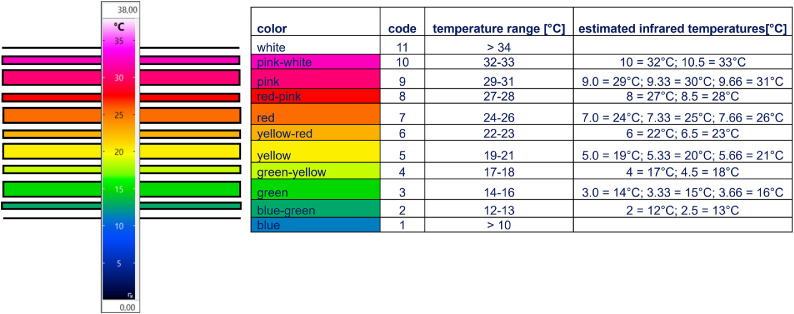
Infrared thermographic image temperatures and corresponding color codes used for the color coded image analysis scoring.
4. **Faecal sampling and analyses:** During examination, individual faeces were sampled as free-catch sample and cooled in a mobile cooling box. Samples were analysed using McMaster method (Gordon & Mc Whitlock, 1939; Whitlock, 1948) with the modified chamber according to Wetzel (1951). 5 g of faecal sample were mixed with 15 ml of saturated saline solution (approximately 360 g sodium chlorine per 1 l of water; density 1.2 g/ml) using a mortar and pestle. The mixture was then sieved through a sieve with 150 µm mesh size into a measuring cylinder. The cylinder was filled up to 60 ml with the saturated saline solution and a stirring bar was added. Afterwards the solution was stirred for 2 min on highest level. A pipette was used to extract 2 ml of solution directly from the central vortex. The first drop was discharged, and the McMaster chamber (Marienfeld-Superior, Art.-Nr. 0611141) was filled. The chamber has a depth of 1.5 mm and 3 counting grids. After a flotation time of two minutes the egg count was performed using a microscope. The number of eggs per gram faeces was calculated using the following formula: EpG = (total number of eggs counted * volume of suspension)/(amount of faeces * size of grid * chamber heights * number of grids). For this study the formula was EPG = (total number of eggs counted * 60 ml)/(5 g * 1 cm^2^ * 0.15 cm *3). Resulting faecal egg counts (FEC) were scored in three categories: low FEC group (≤500 Eggs per Gram faeces), medium FEC group (>500 ≤ 1500 Eggs per Gram faeces) and high FEC group (>1500 Eggs per Gram faeces) (Landesuntersuchungsamt Rheinland-Pfalz, 2018; Taylor, 2010). Only eggs of strongylide species were counted. No species differentiation was performed. Eggs of coccidia were noted but not counted.
5. **Performance data:** In addition, on each examination day, the goat owner provided the most recent milk performance test results. From these, milk yield [kg] and protein content [%] were extracted for further analysis.

### 2.3 Statistical analyses

For statistical analysis SAS® studio (Version 3.8, Enterprise Edition, Institute Inc. Cary, NC, USA) was used.

A linear mixed model (PROC MIXED) was used to analyse the effect of breed on various biological characteristics (e.g. age, animal welfare indicators, linear measurements and performance data; see Table 2). The fixed effect was breed (two levels: GWG, GFG). The parameters were estimated using restricted maximum likelihood (REML), and the degrees of freedom were calculated using the Kenward-Roger method. The significance level was set at α = 0.05.

**Table 2:** Means and standard deviations of the evaluated animal-based parameters, with number of observations and differentiated in the two breeds with *p*-value for differences between breeds. Different superscript letters describe significant differences (*p* < 0.05) within the breeds in one line.

	GFG		GWG		p-value
	Mean ± SD	n	Mean ± SD	n	
**Age** [a]	4.55 ± 2.43	558	4.43 ± 2.33	335	0.4691
**Weight** [kg]	62.25 ± 10.49^a^	547	73.99 ± 11.98^b^	335	**<0.0001**
**Circumference belly [cm]**	112.91 ± 8.84^a^	546	119.09 ± 8.46^b^	335	**<0.0001**
**Circumference chest [cm]**	91.88 ± 5.69^a^	361	98.10 ± 5.34^b^	206	**<0.0001**
**BCS_sternal**	2.82 ± 0.62^a^	544	3.36 ± 0.71^b^	333	**<0.0001**
**BCS_lumbal**	2.49 ± 0.61^a^	362	3.05 ± 0.73^b^	206	**<0.0001**
**Nasal discharge score**	0.00 ± 0.06	547	0.01 ± 0.11	335	0.1467
**Eye discharge score**	0.02 ± 0.13	547	0.01 ± 0.10	334	0.3539
**Faecal soiling score**	0.02 ± 0.14	546	0.01 ± 0.11	331	0.3727
**Coat condition score**	0.04 ± 0.20	546	0.03 ± 0.17	335	0.4218
**Milk yield [kg]**	2.59 ± 1.04^a^	506	2.89 ± 1.22^b^	334	**0.0001**
**Protein [%]**	3.17 ± 0.46^a^	506	3.25 ± 0.38^b^	334	**0.0105**
**Faecal egg count** [eggs per gram faeces]	1298.51 ± 1379.96^a^	525	506.12 ± 821.70^b^	314	**<0.0001**

Another linear mixed model (PROC MIXED) was used to investigate the relationship between the parasite category (three levels: FEC category 1, 2, 3) and various biological characteristics as well as the IRT parameters, separate for each breed. The parasite category was included in the model as a fixed effect. The parameters were estimated using restricted maximum likelihood (REML), and the degrees of freedom were calculated using the Kenward-Roger method. In the case of a significant global effect, pairwise comparisons of the least squares means were performed with Tukey correction (α = 0.05).

## 3 Results

### 3.1 Overview of collected parameters

A total of 893 goats, along with their corresponding faecal samples, were included in the data analysis. The evaluated animal-based indicators nasal discharge, eye discharge, faecal soiling and coat condition served as clinical parameters, but deviations from physiological norms were observed only in individual animals (Table 2). No differences were detected between the breeds regarding these parameters.

Significant differences were detected for body weight, linear measurements (belly and chest circumferences), body condition scores (both sternal and lumbal), performance parameters (milk yield and protein content) and the mean FEC from individual faeces between GFG and GWG (Table 2): GFG had lower body weight, lower circumferences (belly and chest), lower BCS (sternal and lumbal), lower performance data, but higher FEC compared with GWG.

### 3.2 Differences regarding the FEC-categories

None of the clinical parameters (nasal discharge, eye discharge, faecal soiling, and coat condition) showed a significant effect regarding the defined FEC-categories. For age, weight, and BCS (lumbal) as well as for chest circumference and performance parameters (milk yield and protein content) significant differences between FEC-categories were achieved for GFG (see Table 3), but not for GWG (with exception of age). GFG with low FEC had up to 3 kg more body weight, up to 0.3 score points higher BCS, up to 2.75 cm larger chest circumference and up to 0.5 kg higher milk yield and 0.1% higher protein content. GFG belonging to the highest FEC category had the significant lowest milk yield and the significant lowest BCS lumbal (Table 3). For milk yield significant differences were found between each category for GFG, whilst for GWG no difference in milk yield or any parameter (except of age) could be detected (Table 3).

**Table 3:** Results of the mixed model procedure for the physiological parameters differentiated in FEC-categories (category 1 ≤ 500 Eggs per Gram faeces; category 2 > 500 ≤ 1500 Eggs per Gram faeces; category 3 > 1500 Eggs per Gram faeces) and given number of observations, differentiated in the two breeds (GFG= German Fawn Goat; GWG= German White Goat). Different superscript letters describe significant differences (*p* < 0.05) within a line and each breed.

GFG					
	FEC category 1	FEC category 2	FEC category 3	*p*-value	n
**Age [years]**	4.46 ± 0.18^ab^	4.20 ± 0.17^a^	4.97 ± 0.19^b^	**0.0112**	523
**Weight [kg]**	63.93 ± 0.80^a^	61.70 ± 0.75^b^	60.94 ± 0.84^b^	**0.0259**	523
**Circumference belly [cm]**	113.90 ± 0.67	112.37 ± 0.63	112.49 ± 0.71	0.1986	522
**Circumference chest [cm]**	93.38 ± 0.47^a^	91.09 ± 0.51^b^	90.63 ± 0.60^b^	**0.0003**	350
**BCS_sternal**	2.88 ± 0.05	2.82 ± 0.04	2.74 ± 0.05	0.1328	520
**BCS_lumbal**	2.62 ± 0.05^a^	2.48 ± 0.05^a^	2.31 ± 0.06^b^	**0.0008**	351
**Milk yield [kg]**	2.88 ± 0.08^a^	2.56 ± 0.07^b^	2.31 ± 0.08^c^	**<0.0001**	481
**Protein [%]**	3.16 ± 0.04^ab^	3.23 ± 0.03^a^	3.10 ± 0.04^b^	**0.0424**	481

GWG					
	FEC category 1	FEC category 2	FEC category 3	p-value	n
**Age [years]**	4.21 ± 0.15^a^	4.96 ± 0.28^b^	5.48 ± 0.46^b^	**0.0050**	313
**Weight [kg]**	74.00 ± 0.79	74.40 ± 1.42	72.55 ± 2.38	0.7995	313
**Circumference belly [cm]**	119.11 ± 0.56	119.61 ± 1.01	118.10 ± 1.69	0.7409	313
**Circumference chest [cm]**	98.41 ± 0.43	97.22 ± 0.85	96.85 ± 1.52	0.2774	201
**BCS_sternal**	3.34 ± 0.05	3.29 ± 0.09	3.46 ± 0.15	0.6437	311
**BCS_lumbal**	3.06 ± 0.06	2.95 ± 0.12	3.17 ± 0.21	0.5970	201
**Milk yield [kg]**	2.88 ± 0.08	2.72 ± 0.15	2.97 ± 0.25	0.5601	308
**Protein [%]**	3.25 ± 0.03	3.26 ± 0.05	3.23 ± 0.08	0.9456	308

Regarding the thermographic parameters (IRT), no differences could be detected between left and right image sides (Fig. 2(a) and (b)).

The highest infrared thermographic temperatures were recorded at the eye, followed by the udder (see Table 4). The lowest infrared thermographic temperatures were observed at the horns and the lower front and hind legs. For IRT measurements the highest values were recorded mainly in animals belonging to FEC category 3 (see Table 4). Interestingly, ear and horn IRT score were higher for GWG than for GFG, while other parameters showed nearly same scores.

**Table 4:** Results of the mixed model procedure for the infrared thermographic parameters differentiated in FEC-categories (category 1 ≤ 500 Eggs per Gram faeces; category 2 > 500 ≤ 1500 Eggs per Gram faeces; category 3 > 1500 Eggs per Gram faeces) and given number of observations, differentiated in the two breeds (GFG= German Fawn Goat; GWG= German White Goat). Color-code score represents the result of the color scoring (number 1–11) and IRT represents the measured (or corresponding) infrared thermographic temperature. Different superscript letters describe significant differences (*p* < 0.05) within a line and each breed.

	**GFG**							
	**FEC category 1**		**FEC category 2**		**FEC category 3**		*p*-value	**n**
	**color-code score**	**IRT [**°**C]**	**color-code score**	**IRT [**°**C]**	**color-code score**	**IRT [°C]**		
**IRT_eye_temp**		35.61 ± 0.13^ab^		35.29 ± 0.11^a^		35.71 ± 0.12^b^	**0.0222**	304
**IRT_udder_temp**		33.51 ± 0.25		33.48 ± 0.24		34.26 ± 0.26	0.0551	208
**IRT_eye_temp_l**		35.81 ± 0.13^a^		35.35 ± 0.11^b^		35.70 ± 0.12^a^	**0.0159**	274
**IRT_eye_temp_r**		35.68 ± 0.14		35.40 ± 0.11		35.60 ± 0.13	0.2543	265
**IRT_ear_l**	5.60 ± 0.18^a^	∼ 20	5.51 ± 0.18^a^	∼ 20	6.48 ± 0.21^b^	∼ 23	**0.0010**	370
**IRT_ear_r**	5.57 ± 0.19^a^	∼ 20	5.58 ± 0.19^a^	∼ 20	6.50 ± 0.21^b^	∼ 23	**0.0013**	372
**IRT_forehead**	6.32 ± 0.11^a^	∼ 22	6.12 ± 0.11^a^	∼ 22	6.66 ± 0.12^b^	∼ 23	**0.0029**	421
**IRT_horn_l**	5.32 ± 0.23		5.42 ± 0.20		5.87 ± 0.23		0.1888	344
**IRT_horn_r**	5.43 ± 0.22		5.31 ± 0.19		5.89 ± 0.23		0.1400	339
**IRT_body_l**	7.44 ± 0.10^a^	∼ 25	7.52 ± 0.09^a^	∼ 25	8.07 ± 0.10^b^	∼ 27	**<0.0001**	504
**IRT_body_r**	7.40 ± 0.10^a^	∼ 25	7.52 ± 0.09^a^	∼ 25	8.07 ± 0.10^b^	∼ 27	**<0.0001**	505
**IRT_front leg_l**	5.44 ± 0.16^a^	∼ 20	5.42 ± 0.15^a^	∼ 20	5.97 ± 0.17^b^	∼ 21	**0.0324**	504
**IRT_front leg_r**	5.46 ± 0.16^a^	∼ 20	5.43 ± 0.15^a^	∼ 20	5.97 ± 0.17^b^	∼ 21	**0.0291**	506
**IRT_hind leg_l**	5.69 ± 0.16		5.59 ± 0.15		5.97 ± 0.17		0.2242	501
**IRT_hind leg_r**	5.74 ± 0.16		5.56 ± 0.15		5.95 ± 0.17		0.2292	503
**IRT_udder**	9.16 ± 0.17		9.06 ± 0.15		9.04 ± 0.18		0.8516	499
	**GWG**							
	**FEC category 1**		**FEC category 2**		**FEC category 3**		**p-value**	**n**
	**color-code score**	**IRT [**°**C]**	**color-code score**	**IRT [**°**C]**	**color-code score**	**IRT [**°**C]**		
**IRT_eye_temp**		35.94 ± 0.09		35.70 ± 0.14		36.24 ± 0.19	0.0689	142
**IRT_udder_temp**		34.11 ± 0.19		34.31 ± 0.31		34.78 ± 0.48	0.4095	126
**IRT_eye_temp_l**		35.80 ± 0.09		35.67 ± 0.17		36.23 ± 0.27	0.2115	130
**IRT_eye_temp_r**		35.90 ± 0.09		35.82 ±0.16		36.33 ± 0.20	0.0962	132
**IRT_ear_l**	6.26 ± 0.18		6.27 ± 0.31		7.00 ± 0.60		0.4953	206
**IRT_ear_r**	6.24 ± 0.18		6.27 ± 0.31		7.14 ± 0.57		0.3239	210
**IRT_forehead**	6.20 ± 0.11^a^	∼ 22	6.15 ± 0.19^a^	∼ 22	7.11 ± 0.33^b^	∼ 24	**0.0284**	228
**IRT_horn_l**	6.11 ± 0.27		6.38 ± 0.49		6.33 ± 0.92		0.8806	97
**IRT_horn_r**	6.30 ± 0.27		6.65 ± 0.48		6.17 ± 0.93		0.7899	100
**IRT_body_l**	7.23 ± 0.09^a^	∼ 24	7.68 ± 0.16^b^	∼ 26	8.19 ± 0.26^b^	∼ 27	**0.0005**	250
**IRT_body_r**	7.20 ± 0.09^a^	∼ 24	7.65 ± 0.16^b^	∼ 26	8.10 ± 0.27^b^	∼ 27	**0.0015**	248
**IRT_front leg_l**	5.88 ± 0.15		6.08 ± 0.26		6.81 ± 0.44		0.1248	251
**IRT_front leg_r**	5.92 ± 0.15		6.26 ± 0.26		6.90 ± 0.43		0.0739	251
**IRT_hind leg_l**	5.96 ± 0.15		6.20 ± 0.26		6.76 ± 0.44		0.2067	251
**IRT_hind leg_r**	6.12 ± 0.15		6.23 ± 0.26		6.90 ± 0.42		0.2206	249
**IRT_udder**	9.25 ± 0.15		9.34 ± 0.15		9.43 ± 0.15		0.9438	254

For GFG, IRT measurements showed significant differences for FEC category 3, having a significant higher infrared temperature at ear, forehead, lower front leg, and body (Table 4; converted into infrared temperatures up to 3°C difference). Interestingly, for GWG significant differences regarding the FEC categories were only found for forehead (significant differences for FEC category 3; converted into infrared temperatures up to 2 °C difference) and for body (significant difference for FEC category 1 compared with the other categories; converted into infrared temperatures up to 2 °C difference). In all significant cases, the highest infrared temperatures were assessed in animals belonging to the high FEC category (category 3).

## 4 Discussion

The study evaluated clinical parameters, linear parameters, performance parameters and thermographic image analyses in order to develop a practical approach for assessing individual FEC directly on-farm in dairy goats. Interesting results were achieved to categorize animals into FEC categories based on IRT measurements.

### 4.1 Clinical indicators

The examined clinical indicators (Table 2) showed only individual goats having a difference to physiologic parameters. Therefore, it can be stated that all goats in this study were healthy and did not show any signs of clinical infections, detectable via eye or nasal discharge, faecal soiling or coat condition.

Significant differences were evaluated regarding the observed parameters, with GWG having significant higher body weight with larger circumferences and higher BCS than GFG and additionally higher performance data but lower parasitic load (Table 2). These differences in performance data can be attributed to larger body size, including higher body weight, greater circumferences, and possibly higher BCS, as previously noted in the literature (e.g., Gall, 1980).

### 4.2 FEC diagnostic

Faecal egg count (FEC) is served as a selection parameter in animal breeding in different parts of the world (Brown & Fogarty, 2017). As shown in previous studies, parasite burden is typically over-dispersed (Avery et al., 2025; Hoste et al., 2002, 2001; Torres-Acosta et al., 2014). Therefore, it is crucial to develop rapid methods for identifying infected animals and those with lower natural resistance to gastrointestinal parasites. Using these measures can help farmers in performing selective treatment and reduce the amount of anthelmintics used in their herds (Torres-Acosta et al., 2014) as well as selecting animals for breeding. The McMaster method is the most widely used standard quantitative technique for FEC analysis (Gordon & Whitlock, 1939; Whitlock, 1948). But McMaster cannot be used to identify all kinds of parasites, e.g. Haemonchus contortus. As only strongylide eggs were counted in the present study and no species differentiation was performed, the possible influence of different strongylide species on clinical, linear, performance or IRT measurements cannot be evaluated by now. Therefore, in further studies species differentiation should be considered, in order to gain information about different local or systemic host responses.

### 4.3 FEC and clinical, linear and performance parameters

For both breeds, age seems to have an effect on parasite load, as older animals belong to FEC category 3 (Table 3). But, animals in the present study had a mean age of four years therefore age effects should not be overestimated.

For GFG, significant differences were observed across FEC categories in relation to the examined parameters. Animals in FEC category 3 had significantly lower BCS (lumbal; 0.3 score points difference between category 3 and 1) and body weight (2.99 kg difference between category 3 and 1) compared to those in categories 1 and 2. This indicates that a higher parasitic load is associated with a lower BCS and reduced body weight. These results align with previous findings (e.g., Amarante et al., 2004; Sajovitz et al., 2023; Van Wyk et al., 2006), which demonstrated that animals with higher parasite burdens tend to have poorer body condition and lower weight. But the present differences were very small (Table 3), which cannot be detected on-farm by a farmer selecting animals for parasitic treatment (e.g. BCS 2.6 in FEC category 1 and BCS 2.3 in FEC category 3; Table 3). Even the weight difference can be due to food changes or based on reproductive state or lactation period.

Regarding GFG, beside body weight and BCS, significant differences could be detected, as animals with low FEC (category 1) had larger chest circumferences (2.75 cm difference between category 3 and 1) and more milk yield (0.57 kg difference between category 3 and 1) than animals belonging to FEC category 2 or 3. It should be analysed in further studies, whether chest circumference could be a useful tool for selecting animals according to its parasite burden. Chest circumference is already used in animal breeding, shows a good heritability (h^2^ = 0.47, Lange et al., 2018) and might therefore be a useful tool in terms of breeding selection. But, in terms of measuring parasitic load on-farm, chest circumference showed only small differences between the FEC categories in the present study, which might not be usable on-farm with differences up to 2.75 cm (Table 3; GFG). Additionally, it has to be kept in mind, that it might be variable due to reproductive state or during lactation throughout a year.

For milk yield, significant differences were found between each category for GFG. The present findings are in line with the findings of Mavrot et al. (2015), who underlined the influence of parasitic load on milk yield and weight gain in sheep. As this is a herd-specific parameter more analyses have to be made and goat individual tipping points must be defined to be a useful tool, as many factors may affect milk yield.

For GWG no difference in milk yield or any physiological parameter (except of age) could be detected. The different findings in GFG and GWG might be traced back to the differences in parasitic load, as shown in mean eggs per gram for each FEC category (Table 2), resulting in GWG having lower parasitic load. However, these differences are not so large and regarding clinical parameters no differences were detected, although differences between breeds exist in terms of FEC. Maybe the differences between the two breeds can be traced back to the different number of animals or due to a farm effect. In animal breeding, these breeds received nearly same results in performance testing (Herold et al., 2018).

### 4.4 FEC and thermographic parameters

As shown for GFG, several IRT measurements showed significant differences regarding the three analysed FEC categories. Most of the parameters showed the same significant differences regarding the FEC categories, examined from the left or right side of the animal. This leads to the conclusion that (1) measurements were valid and (2) to image and evaluate both sides of a goat seems to be not necessary in this case. This can be confirmed by Scherf et al. (2019), who found symmetrical thermographic patterns between left and right body sides. Therefore, in terms of parasitic diagnostics, body side seems to be not important.

For GFG, nine IRT measurements (regions: eye; and left and right side of ear, forehead, body, lower front leg) allow a differentiation in FEC categories based on the infrared thermographic temperature of the specific region. These results show that animals having a significant higher infrared thermographic temperature in the examined region, belong to the high FEC group with > 1500 eggs per gram faeces. These differences seem to be transferable on-farm, as IRT_ear of FEC category 3 showed a 3 °C difference to the other FEC categories. IRT_body resulted in 2 °C difference, whilst IRT_forehead and IRT_front leg resulted in 1 °C difference. A difference of 3 °C seems to represent a noticeable difference in practical animal selection on-farm. But of course, these findings require further validation and other potential causes of elevated temperatures must be ruled out, such as infections (e.g. subclinical mastitis in dairy cows, as reported by Oliveira et al., 2022) or increased activity (as noted by Ermatringer et al., 2019). This was the case in the present study, where infectious diseases based on the performed clinical scoring and movement, reduced due to the acclimatization time, as a cause for increased temperatures, were excluded as influencing factors.

Interestingly, for GWG only two IRT measurements (forehead and body) allowed a differentiation in FEC categories based on the infrared thermographic temperature of the regions. These differences between GFG and GWG might be explained by the different animal numbers, resulting in different numbers of evaluated parameters or might be due to

a) differences in robustness of parasitic infections. This might therefore be explained by a higher parasitic robustness in GWG, underlined by the fact that no clinical parameter, no BCS variation and no performance reduction (lower milk yield or protein content) was detected, even in animals belonging to FEC category 3 for GWG. Whilst in GFG, animals belonging to category 3 showed (significant) decrease in nearly all evaluated parameters (Table 3). This might also be underlined by the fact that the mean FEC in GFG was significantly higher than in GWG. Reports about differences and resistances in small ruminant breeds are reported (Gauly, 2009), but to our knowledge there is nothing reported about GFG and GWG.
b) differences in parasite species. As animals belong to eight different farms, not all farms and animals of each farm might be infected with the same species. As not all parasites are similar harmful in goats, differences might be explained by different parasite species involved. As no parasite species differentiation was performed in the present study, it cannot be explained whether species differences could result in different thermographic results in GWG compared with GFG. Further data analysis and further studies are needed with parasite species differentiation.
c) differences based on chosen animals and farms. As animal numbers in GWG are smaller than GFG and randomly selected farms differ regarding their parasite management and parasite load, this might have resulted in breed differences which result from animal selection for the present study. As surrounding temperature and humidity affect IRT (Scherf et al., 2019; Church et al., 2014) evaluations on different farms might result in farm-level effects. In the present study, ambient temperatures and humidity’s were assessed at each examination, but did not show large variation within a season. But further analysis of these data, including farm-level effects and parasitic species differentiation should follow. Additionally, results might had benefited from whole herd analyses, but these were not possible due to the time-consuming examinations in the present study. With a reduced number of measurements, this might be possible in further research activities.
d) their different coat colors or different hair densities. Colors, brown and black, in case of GFG or white in case of GWG might result in different thermal conditions, as reported by Magona et al. (2009) or by Scasta (2021) in red, black and white cattle. As in the present study, animal numbers were different between GFG and GWG no correction of color was analysed. As dirt or wet areas might affect thermal imaging (McCafferty, 2007), GWG might be more affected by this as GFG, due to the visibility of dirt and wetting. But interestingly, examined temperature ranges for IRT_forehead and IRT_body were nearly similar comparing both breeds. Therefore, for these two parameters coat color might not be the affecting parameter.

Therefore, several factors might influence the observed differences between GFG and GWG, as well as the general parasitic burden, the different parasitic species, the farm management and the ambient temperature or humidity. These factors were not modelled separately. The aim of this work was a general methodological validation. A detailed analysis should be the subject of further research, including species diagnostics.

### 4.5 Use of infrared thermographic imaging on-farm

When using infrared thermographic (IRT) imaging, several influencing factors must be considered:

a) Weather conditions can affect image quality (Hristov et al., 2008). Increasing ambient temperatures affect IRT parameter, as demonstrated by Scherf et al. (2019) in calves. Therefore, the ambient temperature must be taken into account for IRT measurements, which should be performed in a cool, shadowed area without draughts (Church et al., 2014). In the present study, temperature effects were minimized as possible, as all images were taken indoor or under shelter and temperature varied only in small ranges (12 to 22 °C, respectively 53 to 71 % humidity). Additionally, only dry animals, which had not been exposed to direct sunlight prior to imaging, were included in the present study. This should be taken into account for a validation study, as the experimental condition in the present study have to be transferred into a more practical way, as most dairy goats were kept outdoors, at least part of the daytime.
b) Fur thickness and quality influence surface temperature readings (Bowers et al., 2009). Since all animals were examined during the lactation period, variations in coat characteristics may have affected IRT measurements. This should be analysed in more detail to gain information whether adjusted emissivity might help in this case. As blood flow, hair and skin density as well as surface curvature might interfere with surface temperatures (as discussed e.g. by Wang et al., 2023; McManus et al., 2014; Church et al., 2014). Nonetheless, for the present study, due to the large sample size and the consistent conditions maintained within each farm, this effect is considered negligible.
c) Animal activity prior to imaging can also impact surface temperature (Ermatringer et al., 2019). To control for this, in the present study, all animals were separated from the herd with an acclimatization period of at least five minutes before imaging and all recordings were performed in the same stable to ensure uniformity. This should be taken into account, if imaging is performed on-farm without the experimental setting like in the present study.

Therefore, further research is needed to determine whether IRT can yield comparable results under more practical, on-farm settings such as within the milking parlor to validate the present findings. If imaging directly on-farm without the experimental conditions in the present study, will result in a practical application as a direct health monitoring method for FEC classification, next steps could be a fully automatic installation on-farm.

## Ethics approval

The study was conducted according to the guidelines of the Declaration of Helsinki and was in accordance with national animal welfare law. The animal study protocol was approved by the Institutional Review Board of HfWU (protocol code 2021_14_21.12.21; date of 12 January 2022).

## Declaration of generative AI and AI-assisted technologies in the writing process

The authors did not use any artificial intelligence assisted technologies in the writing process.

## Funding

This work was funded by the Federal Ministry of Agriculture, Food and Regional Identity (BMLEH) based on a decision of the Parliament of the Federal Republic of Germany, granted by the Federal Office for Agriculture and Food (BLE), grant number: 28N-2-035-02.

## CRediT authorship contribution statement

**M. Bernau:** Writing – review & editing, Writing – original draft, Visualization, Supervision, Project administration, Methodology, Conceptualization. **T. Schilling:** Writing – review & editing, Methodology, Investigation. **H. Eßlinger:** Writing – review & editing, Investigation. **L.E. Hoelzle:** Writing – review & editing, Methodology. **S.A. Goth:** Writing – review & editing, Writing – original draft, Visualization, Methodology, Investigation, Formal analysis.

## Declaration of competing interest

Sara Goth and Heiko Eßlinger were researcher during this project and funded by the grant 28N-2-035-02. There are not competing interest.

## Footnotes

⁎ Except of the infrared thermographic goat image and the goat image, all pictograms were license-free images.
